# Transhepatic Insertion of Percutaneous Endoscopic Gastrostomy Tube

**DOI:** 10.1155/2020/4516032

**Published:** 2020-02-12

**Authors:** Zaid Imam, C. Roberto Simons-Linares

**Affiliations:** ^1^Department of Internal Medicine, William Beaumont Hospital, Royal Oak, MI, USA; ^2^Gastroenterology and Hepatology Department, Digestive Diseases Institute, Cleveland Clinic, Cleveland, OH, USA

## Abstract

Inadvertent injury to interposing organs during percutaneous endoscopic gastrostomy (PEG) tube placement is a feared complication of this common and generally safe procedure. Transhepatic PEG insertion is likely an underrepresented complication which may be identified incidentally on imaging or present with life-threatening conditions such as sepsis or massive bleeding. Use of ultrasound in patients with known hepatomegaly may possibly help avoid this complication. We hereby report a case of transhepatic PEG insertion, one of 16 only other cases published in the literature, and review the characteristics of the previous reported cases.

## 1. Introduction

Enteral feeding is the preferred nutrition route for all patients with a functional gastrointestinal (GI) tract as it preserves the tract's physical and functional integrity [1, 2]. Gastrostomy and jejunostomy tubes are the preferred modality for administering enteral nutrition in patients unable to maintain enough oral intake to meet their metabolic needs [3]. PEG placement is preferred over surgical laparoscopic gastrostomy (SLG) placement as risks of general anesthesia are avoided with the former, and a lower risk of minor complications is reported with PEG placement [4].

PEG placement is a generally safe and well-tolerated procedure [5, 6]. Contraindications to PEG placement include severe ascites or coagulopathy, shock, sepsis, abdominal wall infection at the planned gastrostomy site, peritonitis, anatomical, and functional disorders of gastric emptying, and organ interposition [7]. Minor complications related to PEG placement include cutaneous PEG site infections, stomal leakage, and PEG tube dislodgement or obstruction. Life-threatening or severe complications are rare and include gastric perforation, peritonitis, vascular or organ trauma, metastatic tumor spread, necrotizing fasciitis, and buried bumper syndrome [7]. Traversing the liver is an extremely rare but very serious complication of PEG tube placement with only 16 previous case reports in the literature [8–20]. We report a case of transhepatic insertion of a PEG tube in a 92-year-old female incidentally discovered on imaging and review the literature on this complication.

## 2. Case Presentation

A 92-year-old African American female with past medical history significant for toxic megacolon for which she underwent a colectomy with an end ileostomy five years prior was admitted to the hospital for failure to thrive for several months. She had no history of dementia or liver disease, and extensive evaluation revealed no etiology for her condition besides poor intake. A decision to supplement her nutrition enterally was made. She underwent a PEG tube placement after a successful trial of nasogastric feeding.

During her PEG tube placement, her upper endoscopy demonstrated no gastric pathology. A pull method with transillumination was used to insert the PEG tube into position. No immediate complications were noted, and the patient was discharged home.

**Figure 1:**
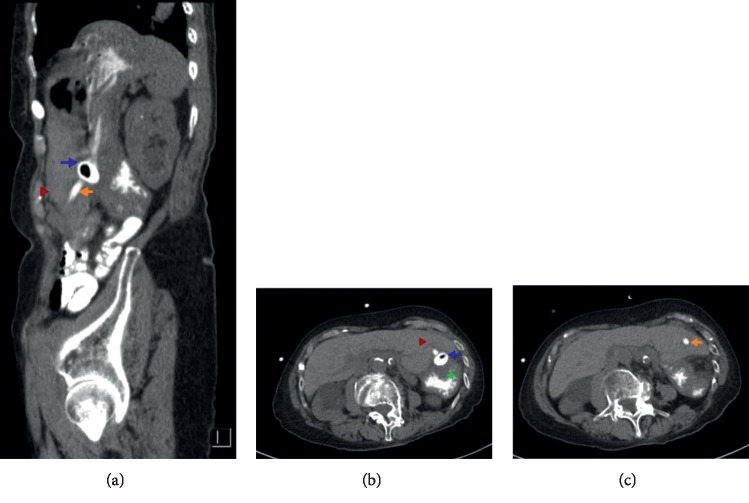
(a–c) Computed tomography of the abdomen demonstrating transhepatic insertion of the PEG tube. Blue arrow: retention balloon in the stomach, orange arrow: PEG tube crossing the left liver lobe, green arrow: stomach, and red arrow: left lobe of the liver.

Two weeks later, she presented with fatigue and abdominal discomfort. On examination, her vital signs were stable, her mucous membranes dry, and her ileostomy and PEG sites were normal. Her initial labs demonstrated a normal white cell count, a hemoglobin of 10.6 g/dl (10.7 g/dl pre-procedure), aspartate aminotransferase of 74 U/L (58 U/L pre-procedure), alanine aminotransferase of 74 U/L (28 U/L before procedure), alkaline phosphatase of 108 U/L (30 U/L before procedure), blood urea nitrogen of 128 mg/dl, and creatinine of 2.45 mg/dl (baseline of 1.2 mg/dl). Intravenous hydration was initiated, and a computed tomography (CT) of the abdomen with oral contrast was obtained which incidentally showed that the PEG tube coursed through the left hepatic lobe with no extravasation of enteric contrast or adjacent hematoma (Figure 1). Given the patient's hemodynamic stability, absence of leak or hematoma, and the need to maintain enteral access given dehydration, a decision to keep the PEG tube in place was done with plans for removal if further complications arose. The PEG tube flushed with no difficulty and was used for enteral nutrition during the patient's inpatient stay.

## 3. Discussion

Gastrostomies can be performed under endoscopic, fluoroscopic, or laparoscopic guidance. The former two techniques avoid complications of general anesthesia and hence are preferred to laparoscopic gastrostomy [5]. A “pull” technique is most commonly used for endoscopic placement of gastrostomy tubes. An alternative technique, the “push” method involves anchoring the stomach prior to PEG insertion with T-fasteners, and the gastrostomy tube is inserted transabdominally rather than transorally.

A comprehensive review of the English literature on inadvertent transhepatic or intrahepatic placement of PEG tubes revealed sixteen other cases, summarized in Table 1. All PEG tubes were inserted using the pull technique except for one. Most of the cases involved insertion through the left hepatic lobe which is expected anatomically. Nine cases (56.3%) complained of abdominal pain, one case (6.25%) presented with tube malfunction, and five cases (31.3%) were discovered incidentally. Three cases (18.8%) had associated bleeding, two of which required liver laceration repairs. Five cases (31.3%) underwent laparotomy. Laparotomy findings included presence of a gastrohepatic fistula in 2 cases, complete intrahepatic migration in 1 case, and a liver laceration in 2 cases. No cases reported hepatic necrosis, a theoretical worrisome complication. The incidence of transhepatic or intrahepatic placement of PEG tubes is likely underreported as most patients report abdominal pain at the PEG site postprocedurally that is frequently attributed to postprocedural abdominal wall pain rather than an ominous complication.

The patient reported had no theoretical risk factors for inadvertent hepatic injury that have been suggested such as hepatomegaly or obesity [20]. A mild otherwise unexplained transaminitis (>2 times upper limit of normal) was detected with no hemoglobin drop. A high suspicion index is needed to detect this complication, and complaints of abdominal pain with transaminitis or hemoglobin changes in a patient even several weeks from a PEG tube placement should warrant careful evaluation for PEG tube-related complications among other pathologies. It is also prudent to evaluate for hepatomegaly with physical examination prior to PEG insertion, and to consider using an ultrasound to evaluate for the presence of interposed liver tissue during PEG placement if hepatomegaly is noted. Additionally, the use of a “push” technique with the use of an anchoring system pulls the stomach closer to the abdominal wall which could displace the left lobe of the liver and hence result in a lower risk of inadvertent transhepatic insertion, supported by the scarcity of this complication being reported with the “push” technique.

In summary, transhepatic insertion of a PEG tube is a serious complication carrying significant morbidity, evidenced by around one-third of patients having to undergo laparotomies as part of the complication's management. Treatment options include keeping the PEG tube in place if it remains functional and no life-threatening complications are noted, or immediate removal if a life-threatening complication such as severe hemorrhage occurs. Concerns with immediate removal would include forming a gastrohepatic fistula. On the other hand, keeping the PEG tube carries the risks of possible migration reported by two cases, and further difficulties with removal. No clear guidelines exist on the management of this complication, and further research is needed to explore the optimal treatment. It seems reasonable, nevertheless, if a transhepatic PEG tube insertion occurs with no hemodynamic changes, significant symptoms, or lab abnormalities, closely observe the patient and continue using the PEG tube.

## Figures and Tables

**Table 1:** Summary of cases of inadvertent transhepatic and intrahepatic PEG placement in the literature.

Author (year)	Age (years)/sex	Indication	Placement technique	Presentation	Diagnosis	Management	Outcome
Stealatto et al (1987) [8]	NR	Gastric decompression; enterocutaneous fistula; perforated sigmoid colon	Pull technique	Incidental	Not reported	No intervention performed to PEG	Died of multiorgan failure 9 days following PEG placement

Stealatto et al (1987) [8]	NR	Gastrointestinal disconnection; subtotal gastrectomy with intra-abdominal sepsis	Pull technique	Incidental	Not reported	No intervention performed to PEG	Gut disconnection resolved and PEG removed

Stealatto et al (1987) [8]	NR	Chronic small bowel obstruction; short gut syndrome	Pull technique	Incidental	Not reported	No intervention performed to PEG	Discharged with PEG tube

Chaer et al (2003) [9]	78, F	Oropharyngeal cancer	Pull technique	Tube malfunction 2.5 months following PEG placement	Computed tomography: intrahepatic PEG	Laparotomy for PEG removal	No long-term complications reported

Gubler et al (2005) [10]	59, M	Nasopharyngeal cancer	Pull technique	Abdominal pain	Ultrasound: PEG tube along the left liver lobe	10-day course of analgesics	Asymptomatic at 6-month follow-up

Gubler et al (2005) [10]	81, F	Esophageal cancer	Pull technique	Abdominal discomfort 1 week following PEG placement	Ultrasound: PEG tube along the left liver lobe edge	3-week course of analgesics	Died at 6 weeks secondary to respiratory failure, thought not PEG-related

Wiggins et al (2007) [11]	61, F	Prolonged mechanical ventilation	Pull technique	Abdominal pain, hypotension 8 hours after procedure	Computed tomography: PEG tube in the left hepatic lobe with 10.1 subcapsular hematoma	Laparotomy: tube removal, repair of liver laceration, and new PEG insertion	Died in 3 months secondary to respiratory failure, thought not PEG-related

Burke et al (2009) [12]	33, M	Intracranial hemorrhage	Pull technique	Fever, chills, and transaminitis 7 weeks after PEG placement	Computed tomography: PEG tube button outside stomach near liver	Antibiotics, laparotomy for tube removal	Long-term outcome not reported

Shaw et al (2009) [13]	35, M	Enteral nutrition in critically ill patient	Pull technique	Abdominal pain 2 days following procedure	Computed tomography: PEG tube inserted through the left liver lobe	Removed 3 months later. No immediate complications reported.	Long-term outcome not reported

Fyock et al (2009) [14]	34, F	Failure to thrive	Not reported	Abdominal tenderness, massive hemorrhage through PEG site	Computed tomography: PEG inserted through the liver	Laparotomy and liver laceration repair	No long-term complications reported

Poggi et al (2013) [15]	56, F	Hypopharyngeal cancer	Pull technique	Abdominal pain, few hours following procedure, mild transaminitis, leukocytosis	Computed tomography: PEG tube inserted through the left liver lobe	Analgesia and antibiotics for few days. PEG removed 4 months later with no complications	Long-term outcome not reported

Mercky et al (2014) [16]	55, F	Squamous cell cancer of the tongue	Pull technique	Abdominal pain 1 week after placement	Computed tomography: intrahepatic PEG placement	Removed. No immediate complications.	No complications at 3-month follow-up

Bichille et al (2014) [17]	57, F	Dysphagia	Pull technique	Incidental, spiked fever of 100.3 F that was drug induced	Computed tomography: PEG tube inserted through the liver	Removed after 1 week. No immediate complications	Long-term outcome not reported

Harta et al (2015) [18]	44, NR	Hypopharyngeal cancer	Pull technique	Abdominal pain, 4 days after placement	Ultrasound: PEG tube in the left hepatic lobe, hepatomegaly	PEG removal and site closure. Reinsertion 1 week later	No immediate complications, long-term outcome not reported

Atalaia-Martins et al (2017) [19]	55, M	Metastatic nasopharyngeal carcinoma	Pull technique	Abdominal pain 1 year after placement	Computed tomography: PEG migrated into the liver with extensive metastasis	Laparotomy and removal of PEG tube	Long-term outcome not reported. No immediate complications

Chhaparia et al (2018) [20]	78, F	Postcardiac arrest, ischemic stroke	Pull technique	Incidental, imaging done to rule out colonic perforation	Computed tomography: PEG in hepatic segment 3, associated 4 cm hematoma	No intervention performed	Died on day 3 of discharge. Thought unrelated to PEG.

M: male, F: female, NR: not reported.

## Supplementary Materials

Supplementary Materials The video legends for the supplementary materials are as follows: Video 1: sagittal computed tomography of the abdomen and pelvis demonstrating transhepatic passage of the percutaneous endoscopic gastrostomy (PEG) tube. Video 2: axial computed tomography of the abdomen and pelvis demonstrating transhepatic passage of the percutaneous endoscopic gastrostomy (PEG tube).
